# Recent advances in autoimmune pancreatitis

**DOI:** 10.3389/fphys.2012.00374

**Published:** 2012-10-02

**Authors:** Terumi Kamisawa, Taku Tabata, Seiichi Hara, Sawako Kuruma, Kazuro Chiba, Atsushi Kanno, Atsushi Masamune, Tooru Shimosegawa

**Affiliations:** ^1^Department of Internal Medicine, Tokyo Metropolitan Komagome HospitalTokyo, Japan; ^2^Department of Gastroenterology, Tohoku UniversitySendai, Japan

**Keywords:** autoimmune pancreatitis, IgG4, international consensus diagnostic criteria, steroid

## Abstract

It is now clear that are two histological types (Type-1 and Type-2) of autoimmune pancreatitis (AIP). The histological pattern of Type-1 AIP, or traditional AIP, is called lymphoplasmacytic sclerosing pancreatitis (LPSP). The histological pattern of Type-2 AIP is characterized by neutrophilic infiltration in the epithelium of the pancreatic duct. In general, Type-2 AIP patients are younger, may not have a male preponderance, and rarely show elevation of serum IgG4 compared with Type-1 AIP patients. Unlike Type-1 AIP patients, Type-2 AIP patients rarely have associated sclerosing diseases, but they are more likely to have acute pancreatitis and ulcerative colitis. Although Type-2 AIP is sometimes observed in the USA and Europe, most AIP cases in Japan and Korea are Type-1. The international consensus diagnostic criteria for AIP comprise 5 cardinal features, and combinations of one or more of these features provide the basis for diagnoses of both Type-1 and Type-2 AIP. Due to the fact that steroid therapy is clinically, morphologically, and serologically effective in AIP patients, it is the standard therapy for AIP. The indications for steroid therapy in AIP include symptoms such as obstructive jaundice and the presence of symptomatic extrapancreatic lesions. Oral prednisolone (0.6 mg/kg/day) is administered for 2–4 weeks and gradually tapered to a maintenance dose of 2.5–5 mg/day over a period of 2–3 months. Maintenance therapy by low-dose prednisolone is usually performed for 1–3 years to prevent relapse of AIP.

## Introduction

Autoimmune pancreatitis (AIP) is a rare disease that has recently emerged as a peculiar type of pancreatitis with a presumed autoimmune etiology. In 1991, Kawaguchi et al. reported two cases of an unusual inflammatory disease involving the pancreas and biliary tract that were resected on suspicion of pancreatic cancer and, based on the peculiar findings of dense infiltration of lymphocytes and plasma cells with marked fibrosis, described the condition as lymphoplasmacytic sclerosing pancreatitis (LPSP) (Kawaguchi et al., 1991). In 1995, Yoshida et al. first proposed the concept of AIP, and they summarized the clinical features as follows: increased serum γ-globulin or IgG levels and the presence of autoantibodies; diffuse irregular narrowing of the main pancreatic duct and enlargement of the pancreas; occasional association with stenosis of the lower bile duct and other autoimmune diseases; mild symptoms, usually without acute attacks of pancreatitis; effectiveness of steroid therapy; and histological finding of LPSP (Yoshida et al., 1995). In 2001, serum IgG4 levels were found to be frequently elevated in AIP patients (Hamano et al., 2001). Since, AIP is frequently associated with various extrapancreatic sclerosing lesions with the same peculiar histological findings as seen in the pancreas, AIP is currently considered to represent a pancreatic lesion of an IgG4-related systemic disease (Kamisawa et al., 2003a,b, 2010c).

Using retrospective, histological examination of pancreases resected on suspicion of pancreatic cancer from patients with mass-forming chronic pancreatitis, American and European pathologists have described another unique histological pattern, called idiopathic duct-centric pancreatitis (IDCP) (Notohara et al., 2003), or AIP with granulocytic epithelial lesion (GEL) (Zamboni et al., 2004). Recently, LPSP has been called Type-1 AIP, and IDCP has been called Type-2 AIP (Sah et al., 2010). This review focuses on the differences between Type-1 and Type-2 AIP, the recently proposed international consensus diagnostic criteria for AIP (Shimosegawa et al., 2011), and the Japanese standard therapeutic regimen for AIP.

## Two subtypes of autoimmune pancreatitis: type-1 and type-2

**Figure 1:**
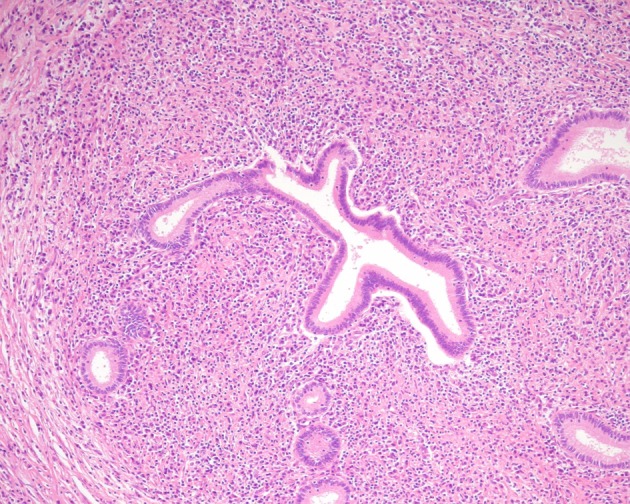
**Histological feature of the pancreas of Type-1 AIP**.

The pathological features of Type-1 and Type-2 AIP differ clearly. LPSP of Type-1 AIP consists of dense infiltration of lymphocytes and IgG4-positive plasma cells and fibrosis that involves pancreatic lobules, ducts and peripancreatic adipose tissue (Kawaguchi et al., 1991; Kamisawa et al., 2003a,b, 2010c). Storiform fibrosis, a swirling pattern of fibrosis mixed with inflammatory cells, and obliterative phlebitis are characteristic and diagnostic (Figure 1). Eosinophils are often present, but neutrophils are absent. Neutrophilic infiltration within the lumen and epithelium of interlobular ducts is a characteristic feature of IDCP of Type-2 AIP (Notohara et al., 2003; Zamboni et al., 2004). Infiltration of IgG4-positive plasma cells is rare (Kamisawa et al., 2010a; Shimosegawa et al., 2011).

Since no apparent serological markers have yet been found for Type-2 AIP, histological examination is necessary to diagnose Type-2 AIP, and thus its clinical diagnosis is difficult. Although the clinical features of Type-2 AIP are not as well-known as those of Type-1 AIP, Type-2 AIP patients show quite different clinical profiles from Type-1 AIP patients. Age at diagnosis of Type-2 AIP is more than a decade younger, and Type-2 AIP does not show a male preponderance. Type-2 AIP patients are less likely to show elevated serum IgG4 levels and IgG4-related extrapancreatic sclerosing lesions such as proximal sclerosing cholangitis, sclerosing sialadenitis, and retroperitoneal fibrosis. Type-2 AIP is more likely associated with acute pancreatitis and inflammatory bowel disease. Type-2 AIP tends to have focal features and is more commonly surgically resected due to the diagnostic difficulty. Although endocrine and exocrine functions of the pancreas are frequently impaired in Type-1 AIP, the functions in Type-2 AIP are unknown. Both Type-1 and Type-2 AIP respond well to steroids, but relapse of Type-2 AIP is rarely seen, whereas Type-1 sometimes relapses (Notohara et al., 2003; Zamboni et al., 2004; Kamisawa et al., 2010a; Sah et al., 2010; Shimosegawa et al., 2011). The prevalence of Type-1 and Type-2 AIP differ around the world. In Japan and Korea, most AIP cases are Type-1, and Type-2 AIP is quite rare (Kamisawa et al., 2011). However, Type-2 AIP may comprise about 20–40% of AIP cases in the United States of America and Europe (Notohara et al., 2003; Zamboni et al., 2004; Park et al., 2009). Whether these differences are induced by regional or racial elements or low recognition of the disease remains unclear.

## International consensus diagnostic criteria

The international consensus diagnostic criteria for AIP (Shimosegawa et al., 2011) were developed to be applicable worldwide, diagnose AIP safely, avoid misdiagnosis of pancreatic cancer as AIP, and diagnose AIP in acute presentation. Criteria for Type-1 and Type-2 AIP were developed separately. The diagnosis of AIP is made by a combination of one or more of five cardinal features: (1) imaging features of the pancreatic parenchyma and pancreatic duct; (2) serology; (3) other organ involvement (OOI); (4) histology of the pancreas; and (5) an optional criterion of response to steroid therapy. Each feature has been categorized as level 1 and 2 findings depending on the diagnostic reliability. The diagnoses of Type-1 and Type-2 AIP can be definitive or probable, and in some cases, the distinction between the subtypes may not be possible (AIP-not otherwise specified). Diagnosis of sero-negative Type-1 AIP is sometimes difficult.

Cross-sectional pancreatic imaging on CT or MRI is considered as the first essential clue, and the findings have been divided into typical diffuse enlargement and indeterminate images of segmental or focal enlargement of the pancreas. For the criterion related to the pancreatic duct on endoscopic retrograde pancreatography (ERP), level 1 is long or multiple narrowings without marked upstream dilatation, and level 2 is segmental or focal narrowing without marked upstream dilatation. For the serological criterion for Type-1 AIP, level 1 is marked elevation of serum IgG4 levels (more than double upper limit of normal value), and level 2 is mild elevation of serum IgG4 levels. For the criterion of OOI for Type-1 AIP, level 1 is either histological findings in extrapancreatic organs (any three of the four features) or typical radiological evidence (proximal bile duct stricture or retroperitoneal fibrosis), and level 2 is histological findings or physical or radiological evidence (symmetrically enlarged salivary/lacrimal glands or renal involvement). For the histological criterion for Type-1 AIP, level 1 is LPSP with more than three features on core biopsy or resected specimens, and level 2 is any two features on core biopsy specimens. A diagnostic steroid trial is an optional criterion. Response to steroid therapy is defined as rapid (within 2 weeks), radiologically demonstrable resolution or marked improvement in pancreatic or extrapancreatic manifestations. However, a steroid trial should be used only after a negative work-up for cancer, including endoscopic ultrasound-guided fine needle aspiration (Table 1). For the criteria for Type-2 AIP, there is no serological criterion; the criterion of OOI is only level 2 (clinically diagnosed inflammatory bowel disease); and the histological criterion is granulocytic infiltration and absent or scant IgG4-positive cells.

**Table 1:** **Level 1 and level 2 criteria for Type-1 AIP**.

**Criterion**	**Level 1**	**Level 2**
Parenchymal imaging	Typical: diffuse enlargement with delayed enhancement (sometimes associated with rim-like enhancement)	Indeterminate (including atypical): segmental/focal enlargement with delayed enhancement
Ductal imaging (ERP)	Long (>1/3 length of the main pancreatic duct) or multiple strictures without marked upstream dilatation	Segmental/focal narrowing without marked upstream dilatation (duct size, <5 mm)
Serology	IgG4, >2x_ upper limit of normal value	IgG4, 1–2x_upper limit of normal value
Other organ involvement (OOI)	a or b	a or b
	a. Histology of extrapancreatic organs	a. Histology of extrapancreatic organs including endoscopic biopsy of bile duct
	Any three of the following:	Both of the following:
	(1) Marked lymphoplasmacytic infiltration with fibrosis and without granulocytic infiltration	(1) Marked lymphoplasmacytic infiltration with fibrosis without granulocytic infiltration
	(2) Storiform fibrosis granulocytic infiltration	(2) Abundant (>10 cells/HPF) IgG4-positive cells
	(3) Obliterative phlebitis	
	(4) Abundant (>10 cells/HPF) IgG4-positive cells	
	b. Typical radiological evidence	b. Physical or radiological evidence
	At least one of the following:	At least one of the following:
	(1) Segmental/multiple proximal (hilar/intrahepatic) or proximal and distal bile duct stricture	(1) Symmetrically enlarged salivary/lacrimal glands
	(2) Retroperitoneal fibrosis	(2) Radiological evidence of renal involvement described in association with AIP
Histology of the pancreas	LPSP (core biopsy/resection)	LPSP (core biopsy)
	At least 3 of the following:	Any 2 of the following:
	(1) Periductal lymphoplasmacytic infiltrate without granulocytic infiltration	(1) Periductal lymphoplasmacytic infiltrate without granulocytic infiltration
	(2) Obliterative phlebitis	(2) Obliterative phlebitis
	(3) Storiform fibrosis	(3) Storiform fibrosism
	(4) Abundant (>10 cells/HPF) IgG4-positive cells	(4) Abundant (>10 cells/HPF) IgG4-positive cells

Diagnostic steroid trial. Response to steroid (Rt) Rapid (e2 wk) radiologically demonstrable resolution or marked improvement in pancreatic/extrapancreatic manifestations.

Definitive Type-1 can be diagnosed only by histological examination of resected pancreas or core biopsy specimens showing LPSP. To diagnose definitive Type-1, in cases with diffuse imaging findings, any additional non-ductal cardinal criterion is necessary. In cases with segmental or focal imaging, two or more from any level 1 and ductal level 2 cardinal criteria are necessary. To make the diagnosis in association with response to steroid therapy, one non-ductal level 1 or ductal level 1 with any non-ductal level 2 cardinal criterion is necessary. Response to steroid with one non-ductal level 2 cardinal criterion is diagnosed as probable Type-1 AIP (Table 2).

**Table 2:** **Diagnosis of definitive and probable Type-1 AIP using international consensus diagnostic criteria**.

**Diagnosis**	**Primary basis for diagnosis**	**Imaging evidence**	**Collateral evidence**
Definitive Type-1 AIP	Histology	Typical/indeterminate	Histologically confirmed LPSP (level 1 H)
	Imaging	Typical	Any non-D level 1/level 2
		Indeterminate	Two or more from level 1 (+level 2 D^*^)
	Response to steroid	Indeterminate	Level 1 S/OOI + Rt or level 1 D + level 2 S/OOI/H + Rt
Probable Type-1 AIP		Indeterminate	Level 2 S/OOI/H + Rt

* Level 2 D is counted as level 1 in this setting.

To diagnose definite Type-2 AIP, histologically confirmed IDCP or clinical inflammatory bowel disease with level 2 histology and response to steroid is necessary.

## Japanese standard steroid treatment for AIP

Steroid therapy is clinically, morphologically, and serologically effective in AIP patients, and as a result, it has become the standard current therapy for AIP. From the international survey of AIP (Kamisawa et al., 2011), steroid therapy is standard for AIP in all countries. According to the Japanese consensus guideline for the management of AIP (Kamisawa et al., 2010b), the indications for steroid therapy in AIP include symptoms such as obstructive jaundice and the presence of symptomatic extrapancreatic lesions. Before steroid therapy, obstructive jaundice should be controlled by biliary drainage and blood glucose levels should be regulated, usually by administration of insulin in diabetes mellitus patients. The initial recommended dose of oral prednisolone for induction of remission is 0.6 mg/kg/day, administered for 2–4 weeks. Biochemical and serological blood tests, such as liver enzyme and IgG4 levels, as well as imaging tests, such as CT, MRCP, and ERCP, are performed periodically after the start of steroid therapy. Pancreatic size usually normalizes within a few weeks, and biliary drainage becomes unnecessary within about 1 month. Rapid response to steroids is reassuring and confirms the diagnosis of AIP. If steroid effectiveness is reduced, the patient should be re-evaluated on suspicion of pancreatic cancer. The dose is gradually tapered to a maintenance dose of 2.5–5 mg/day over a period of 2–3 months.

Remission is defined as, the disappearance of clinical symptoms and resolution of the pancreatic and/or extrapancreatic manifestations on imaging studies. In the Japanese multicenter survey of steroid therapy for AIP (Kamisawa et al., 2009), the remission rate of AIP patients treated with steroid was 98% (451/459). At remission, the enlarged pancreas returned to near-normal size in 239 (80%) of 300 patients and became atrophic in 58 patients (20%). Elevated serum IgG4 levels decreased in all patients after the start of steroid therapy, but they failed to normalize in 115 (63%) of 182 patients. HbA1c and impaired pancreatic exocrine function improved and normalized in half of them.

**Figure 2:**
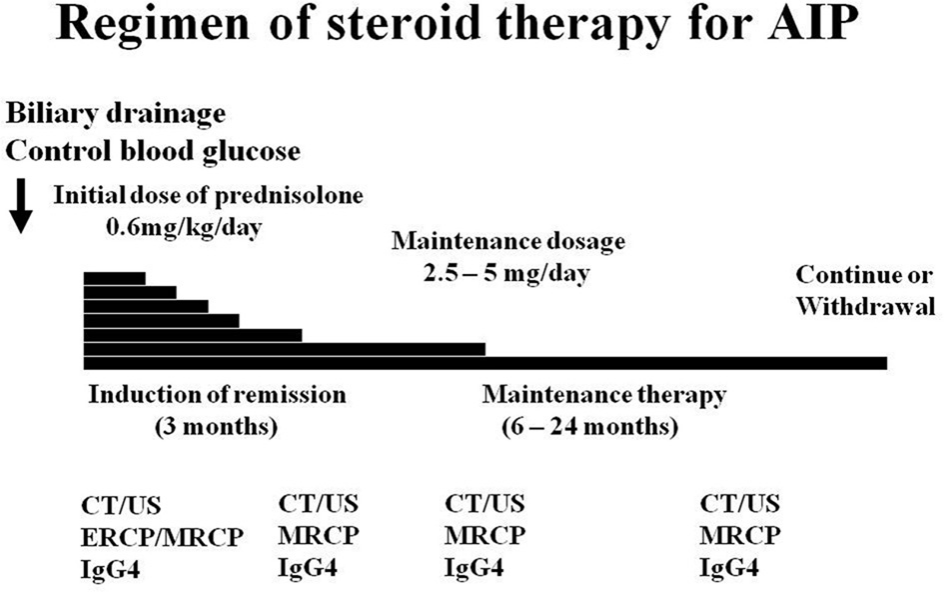
**Regimen of Japanese standard steroid treatment for AIP**.

Relapse of AIP is defined as reappearance of symptoms accompanied by the reappearance of pancreatic and/or extrapancreatic, including bile duct, salivary gland, and retroperitoneal abnormalities on imaging and/or elevation of serum IgG4 levels. In a multicenter survey (Kamisawa et al., 2009), the relapse rate of AIP patients treated with steroid was 24% (110/451). Relapse occurred in the pancreas (*n* = 57, 52%), bile duct (*n* = 37, 34%), and extrapancreatic lesions (*n* = 19). Maintenance steroid therapy was given after remission in 377 (82%) of 459 patients treated with steroid. The maintenance oral prednisolone dose was 10 mg/day (*n* = 27, 7%), 7.5 mg/day (*n* = 13, 3%), 5 mg/day (*n* = 238, 63%), 2.5 mg/day (*n* = 78, 21%), and others. The relapse rate with maintenance therapy was 23% (63/273), which was significantly lower than that of patients who stopped maintenance therapy (34%, 35/104; *p* < 0.05). In the USA and UK, where no maintenance therapy was given, relapse rates of patients treated with steroid were reportedly 38% (Sandanayake et al., 2009) and 53% (Ghazale et al., 2008). Whether maintenance therapy benefits AIP patients remains unconfirmed, but given these findings, maintenance therapy with low dose prednisolone (2.5–5 mg/day) was recommended to prevent relapse (Kamisawa et al., 2009, 2010b). In a multicenter study (Kamisawa et al., 2009), of the 377 patients who underwent maintenance therapy, maintenance therapy was stopped in 104 (28%) in whom complete radiological and serological improvement was achieved. As to the period from the start of steroid therapy to relapse, 32% (32/99) relapsed within 6 months, 56% (55/99) relapsed within 1 year, 76% (75/99) relapsed within 2 years, and 92% (91/99) relapsed within 3 years after starting medication. Maintenance therapy should be stopped within 1–3 years in cases with radiological and serological improvement to prevent steroid-related complications such as osteoporosis, diabetes, and infection (Kamisawa et al., 2009, 2010b) (Figure 2).

For relapsed AIP, re-administration or dose-up of steroid was effective. In the USA and UK, immunomodulatory drugs such as azathioprine were used in addition to re-administered steroid for relapsed patients, and remission was again achieved and maintained on long-term azathioprine (Ghazale et al., 2008; Sandanayake et al., 2009).

It has been reported that the predictors for relapse are the presence of proximal bile duct stenosis and elevated serum IgG4 levels (Ghazale et al., 2008; Kamisawa et al., 2009; Sah et al., 2010; Takuma et al., 2011). Pancreatic stones are formed in relapsing AIP patients, which might be induced by pancreatic juice stasis from intensified incomplete obstruction of the pancreatic duct system (Takuma et al., 2011; Maruyama et al., 2012). Since, AIP might transform into ordinary chronic pancreatitis after several relapses, relapses of AIP should be avoided as much as possible.

It is necessary to verify the validity of the Japanese regimen of steroid therapy for AIP: the necessity, drugs, and duration of maintenance therapy need to be clarified by prospective studies.

## Conclusions

Type-1 and Type-2 AIP are clinicopathologically different entities. International consensus diagnostic criteria can be used to diagnose Type-1 and Type-2 AIP. Steroid therapy is standard therapy for AIP, but the regimen should be evaluated in prospective trials.

### Conflict of interest statement

The authors declare that the research was conducted in the absence of any commercial or financial relationships that could be construed as a potential conflict of interest.
